# Embryos of Robertsonian Translocation Carriers Exhibit a Mitotic Interchromosomal Effect That Enhances Genetic Instability during Early Development

**DOI:** 10.1371/journal.pgen.1003025

**Published:** 2012-10-25

**Authors:** Samer Alfarawati, Elpida Fragouli, Pere Colls, Dagan Wells

**Affiliations:** 1 Nuffield Department of Obstetrics and Gynaecology, University of Oxford, Oxford, United Kingdom; 2 Reprogenetics UK, Institute of Reproductive Sciences, Oxford, United Kingdom; 3 Reprogenetics, Livingston, New Jersey, United States of America; University College London, United Kingdom

## Abstract

Balanced chromosomal rearrangements represent one of the most common forms of genetic abnormality affecting approximately 1 in every 500 (0.2%) individuals. Difficulties processing the abnormal chromosomes during meiosis lead to an elevated risk of chromosomally abnormal gametes, resulting in high rates of miscarriage and/or children with congenital abnormalities. It has also been suggested that the presence of chromosome rearrangements may also cause an increase in aneuploidy affecting structurally normal chromosomes, due to disruption of chromosome alignment on the spindle or disturbance of other factors related to meiotic chromosome segregation. The existence of such a phenomenon (an inter-chromosomal effect—ICE) remains controversial, with different studies presenting contradictory data. The current investigation aimed to demonstrate conclusively whether an ICE truly exists. For this purpose a comprehensive chromosome screening technique, optimized for analysis of minute amounts of tissue, was applied to a unique collection of samples consisting of 283 oocytes and early embryos derived from 44 patients carrying chromosome rearrangements. A further 5,078 oocytes and embryos, derived from chromosomally normal individuals of identical age, provided a robust control group for comparative analysis. A highly significant (P = 0.0002) increase in the rate of malsegregation affecting structurally normal chromosomes was observed in association with Robertsonian translocations. Surprisingly, the ICE was clearly detected in early embryos from female carriers, but not in oocytes, indicating the possibility of mitotic rather than the previously suggested meiotic origin. These findings have implications for our understanding of genetic stability during preimplantation development and are of clinical relevance for patients carrying a Robertsonian translocation. The results are also pertinent to other situations when cellular mechanisms for maintaining genetic fidelity are relaxed and chromosome rearrangements are present (e.g. in tumors displaying chromosomal instability).

## Author Summary

Translocations involve exchange of material between two or more chromosomes and are a common form of genetic abnormality. The rearrangements are difficult to process during meiosis, frequently producing gametes with missing/extra pieces of the affected chromosomes. It has been suggested that translocations might also disrupt the segregation of structurally normal chromosomes, a so-called interchromosomal effect (ICE), but the published data is contradictory. Here we report results from a unique collection of samples, consisting of oocytes and embryos from translocation carriers. Examination of more than 210,000 chromosomes revealed no evidence of an ICE in oocytes, but a significant effect in embryos tested three days after fertilization (6–10 cell stage) in a subset of patients. Clinically, this means that some translocation carriers are at even higher risk of chromosomally abnormal pregnancies than previously suspected, a factor that should be considered during genetic counselling. Scientifically, the results illuminate a poorly understood stage of human development, characterized by chromosomal instability, reminiscent of that observed in some tumors. The restriction of the ICE to a narrow developmental window was unexpected, yet may explain why some earlier studies could not agree on the existence of an ICE.

## Introduction

The incidence of balanced chromosomal rearrangements in the general population is appreciable, detected in 0.19% of newborns [1]. Most carriers of a balanced chromosome rearrangement do not display an obvious phenotype and remain undetected until they attempt to reproduce. The presence of a rearrangement leads to unusual pairing configurations between the derivative chromosomes and their structurally normal homologues during meiosis. This increases the risk of abnormal chromosome segregation and the production of gametes with losses and/or gains of chromosomal material, associated with problems such as miscarriage, birth of children with congenital abnormalities and, in some cases, reduced fertility [2]. Not surprisingly, translocation carriers are found at increased frequency in certain patient populations, such as couples with recurrent miscarriage, where the incidence is 25-fold higher than the general population [3], [4]. The rate is also elevated amongst infertile couples who have had several unsuccessful cycles of in vitro fertilisation (IVF) treatment, affecting 1.4% [5].

It has been suggested that, besides the direct effect on the chromosomes involved in the rearrangement, there may also be an impact on the segregation of other, structurally normal, chromosomes during meiosis. This might be a consequence of disrupted chromosome alignment on the spindle, or due to interference with other key aspects of the chromosome segregation process, leading to a generalised increase in the risk of producing aneuploid gametes. This phenomenon is known as an inter-chromosomal effect (ICE) [6]. Many researchers have sort to establish whether or not an ICE truly exists, but the limitations of the available cytogenetic technologies has meant that conclusive data has remained elusive and the existence of an ICE remains the subject of debate [7]–[17].

Particularly valuable data concerning the possibility of an ICE has come from cases of preimplantation genetic diagnosis (PGD) carried out for carriers of chromosome rearrangements. This process involves the generation of embryos using assisted reproductive technologies followed by genetic analysis. In most instances the embryos develop in vitro until approximately the 8-cell stage, at which time a single cell is removed and analysed using fluorescence in situ hybridisation (FISH) in order to determine the copy number of chromosomal regions involved in the translocation. Only embryos found to be normal/balanced for these regions are transferred to the mother's uterus and consequently the risk of any resulting pregnancy miscarrying or producing a child affected by a congenital abnormality is greatly reduced. The first clinical application of PGD techniques for translocation carriers took place in the late 1990s [18], [19] and since then thousands of PGD cycles have been performed [20].

A focus on gametes and preimplantation embryos is especially valuable when attempting to determine the presence or absence of an ICE since, at early stages of development, elimination of embryos harbouring lethal chromosomal anomalies (via developmental arrest, implantation failure or miscarriage) has not yet taken place. Consequently, the primary incidence of chromosome malsegregation can be assessed. Some previous studies, investigating embryos produced by carriers of chromosome rearrangements undergoing PGD, have produced data supporting the existence of an ICE [8], [12]. However, once again there is controversy with other research suggesting that an ICE is either entirely absent or negligible in these patients [15]. Studies conducted on sperm provide the strongest data in favour of an ICE (see [11] for detailed summary), although the results are variable, with the effect detected in some samples but not others [9], [11]. We are not aware of any studies assessing the possibility of an ICE in female meiosis.

**Figure 1:**
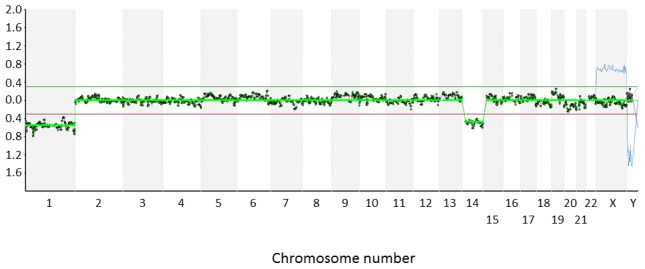
Microarray-CGH analysis of an embryo from a Robertsonian translocation carrier. Cytogenetic analysis of a cleavage stage embryo from a Robertsonian translocation carrier- 46,XY,t(13;15)(q21.3;q11.2). Microarray-CGH revealed monosomy 13, presumably resulting from a meiotic error due to problems processing the rearranged chromosomes. An additional aneuploidy unrelated to the Robertsonian translocation (monosomy for chromosome 1) was also detected. The two monosomies are indicated by the altered ratio of fluorescence related to the test (embryo) and reference (46,XY) DNA samples. All of the probes corresponding to chromosomes 1 and 13 have test/reference ratios less than 0.3.

Very recently, comprehensive chromosomal screening strategies, such as microarray comparative genomic hybridization (aCGH), have begun to replace FISH for the PGD of translocations and other chromosomal rearrangements (Figure 1). Not only do methods of this kind reveal abnormalities affecting the specific chromosomes involved in the rearrangement, but they also detect aneuploidies affecting any other chromosomes [21]–[24]. In contrast, FISH methods only permit a very limited chromosomal screening and consequently most attempts to identify an ICE in human gametes or embryos have been restricted to the analysis of small numbers of chromosomes (additional to those involved in the translocation). While this limitation does not invalidate the results of such studies, it means that very few chromosome segregations can be examined in each sperm or embryo tested.

The aim of this study was to determine whether an ICE truly exists. The strategy was to exploit unique access to a large number of human oocyte and embryo samples derived from carriers of chromosomal rearrangements, coupled with the latest molecular cytogenetic methods. This allowed us to look at more than 10,000 individual chromosomes in samples from chromosome rearrangement carriers and more than 200,000 chromosomes in well-matched controls. The large number of chromosomes evaluated greatly increased the sensitivity and statistical power of this investigation compared with preceding studies. The study revealed that an ICE does exist, but suggests that it may be confined to a narrow developmental window and may be associated with specific types of chromosomal rearrangement. These results were unexpected and yet may explain some of the apparently contradictory findings in previous studies. Additionally, the findings raise important questions concerning fundamental aspects of cell biology and have implications for the clinical management of patients with chromosome rearrangements.

## Results/Discussion

Overall, a total of 283 samples derived from chromosome rearrangement carriers were analyzed (Table 1 and Table 2). Oocytes were assessed by comprehensive chromosome analysis of the first and second polar bodies, cleavage stage embryos were evaluated following removal of a single cell, while analysis of blastocysts involved the biopsy and testing of an average of five cells per embryo. A robust control group was created for each patient by careful matching with data from oocytes, cleavage stage embryos or blastocysts from multiple individuals of normal karyotype and identical female age, having treatment at the same clinic during the same time interval and using the same diagnostic test (i.e. microarray-CGH). The chromosomally normal control patients had requested oocyte or embryo testing as part of a routine IVF cycle, with the aim of reducing the risk of miscarriage and Down syndrome caused by spontaneously arising aneuploidy. The control group consisted of 5,078 samples.

**Table 1:** Patient information.

Patient number	Rearrangement	Stage tested
**1**	46,XX,t(9;16)(p13.1;p11.2)	PB
**2**	46,XY,t(17;19)(q25.3;q13.1)	Bla/Bla
**3**	46,XY,t(16;17)(q11;p13.3)	Bla
**4**	45,XY,der(13;14)(q10;q10)	TE/TE
**5**	46,XY,t(1;2)(q23.1;q35)	Bla/TE
**6**	45,XY,der(14;21)(q10;q10)	TE
**7**	45,XY,der(13;14)(q10;q10)	Bla
**8**	46,XY,t(1;3)(q31;p13)	TE
**9**	45,XY,der(14;21)(q10;q10)	Bla
**10**	46,XY,t(5;15)(q13.1;q21.2)/46,XX,inv(3)(q25.1;q26.2)	Bla
**11**	46,XY,t(11;22)(q23;q11.2)	TE
**12**	45,XY,der(13;15)(q10;q10)	TE
**13**	46,XY,t(13;15)(q21.3;q11.2)	Bla
**14**	45,XX,der(13;15)(q10;q10)	Bla
**15**	46,XX,t(17;18)(p11.2;p11.2)	Bla
**16**	46,XX,t(8;9)(p12;q31)	TE
**17**	46,XY,t(1;8)(q25.3;p11.2)	Bla
**18**	46,XX,t(10;16)(q11.2;p11.2)	Bla
**19**	46,XX,t(1;2)(q24;p21)	Bla/TE
**20**	45,XY,der(13;14)(q10;q10)	Bla
**21**	46,XX,t(1;5)(p36.1;q33)	TE/TE
**22**	46,XY,inv(8)(p21q24.1)	TE
**23**	46,XY,t(10;17)(q21.2;p11.2)	Bla
**24**	45,XY,der(13;14)(q10;q10)	Bla
**25**	46,XY,t(7;18)(p15.3;q12.2)	Bla
**26**	45,XY,der(13;14)(q10;q10)	Bla
**27**	45,XX,der(13;14)(q10;q10)	Bla
**28**	45,XX,der(13;14)(q10;q10)	PB
**29**	45,XX,der(13;21)(q10;q10)	Bla/Bla
**30**	46,XY,t(1;3)(q31;p13)	Bla
**31**	45,XX,der(13;14)(q10;q10)	Bla
**32**	45,XY,der(14;21)(q10;q10)	Bla
**33**	45,XY,der(14;21)(q10;q10)	Bla
**34**	46,XY,t(17;21)(q21;q22)	Bla
**35**	46,XY,t(1;15)(q21p11.2)	Bla
**36**	46,XX,t(7;10)(p11.2;p11.23)	Bla
**37**	46,XY,t(5;7)(q23.2;p14)	TE
**38**	45,XX,der(13;14)(q10;q10)	TE
**39**	45,XX,der(13;14)(q10;q10)	PB/PB/TE
**40**	46,XX,t(4;11)(q24;q11)	Bla
**41**	46,XX,inv(5)(p13.1–q13.3)	PB/TE
**42**	45,XX,der(13;14)(q10;q10)	PB/PB
**43**	46,XX,t(9;16)(p13.1;p11.2)	PB
**44**	45,XX,der(13;21)(q10;q10)	PB

Bla: Blastomere; PB: polar body; TE: trophectoderm cells; aCGH: Microarray-CGH; Met-CGH: Metaphase CGH. Note: Some patients underwent more than one cycle of PGD and may have had analysis conducted at different stages in subsequent cycles.

**Table 2:** Number of embryos from each cycle and the number of chromosomes assessed along with abnormalities detected.

Patient	Case	Maternal age	Type of rearrangement	Stage tested	Embryos/oocytes with a result	Number of chromosomes assessed*	Number of errors detected**
**1**	1	26	Rec	PB	3	63	1
**2**	2	29	Rec	Bla	7	294	7
**2**	3	30	Rec	Bla	11	462	6
**3**	4	30	Rec	Bla	5	210	17
**4**	5	31	Rob	TE	13	546	9
**4**	6	31	Rob	TE	9	378	2
**5**	7	31	Rec	TE	3	126	0
**6**	8	31	Rob	TE	2	84	4
**7**	9	32	Rob	Bla	4	168	10
**5**	10	32	Rec	Bla	5	210	13
**8**	11	32	Rec	TE	7	294	13
**9**	12	33	Rob	Bla	2	84	4
**10**	13	33	Rec/Inv	Bla	8	320	37
**11**	14	33	Rec	TE	9	378	2
**12**	15	34	Rob	TE	11	462	7
**13**	16	34	Rec	Bla	3	126	2
**14**	17	34	Rob	Bla	7	294	22
**15**	18	34	Rec	Bla	3	126	1
**16**	19	34	Rec	TE	5	210	3
**17**	20	35	Rec	Bla	2	84	2
**18**	21	35	Rec	Bla	5	210	5
**19**	22	35	Rec	Bla	3	126	24
**20**	23	35	Rob	Bla	7	294	23
**21**	24	35	Rec	TE	5	210	1
**21**	25	35	Rec	TE	4	168	2
**22**	26	36	Inv	TE	3	132	1
**23**	27	36	Rec	Bla	3	126	4
**24**	28	36	Rob	Bla	2	84	10
**25**	29	36	Rec	Bla	4	168	3
**26**	30	36	Rob	Bla	5	210	18
**27**	31	36	Rob	Bla	8	336	43
**19**	32	36	Rec	TE	2	84	0
**28**	33	36	Rob	PB	4	84	3
**29**	34	37	Rob	Bla	6	252	22
**29**	35	37	Rob	Bla	5	210	5
**30**	36	37	Rec	Bla	9	378	29
**31**	37	37	Rob	Bla	6	252	29
**32**	38	38	Rob	Bla	2	84	2
**33**	39	39	Rob	Bla	8	336	27
**34**	40	39	Rec	Bla	2	84	4
**35**	41	40	Rec	Bla	7	294	10
**36**	42	40	Rec	Bla	3	126	3
**37**	43	41	Rec	TE	3	126	4
**38**	44	41	Rob	TE	5	210	17
**39**	45	42	Rob	PB	9	189	17
**39**	46	42	Rob	PB	8	168	16
**39**	47	42	Rob	TE	3	126	4
**40**	48	42	Rec	Bla	5	210	22
**41**	49	43	Inv	PB	7	154	12
**41**	50	43	Inv	TE	2	88	2
**42**	51	36	Rob	PB	4	84	3
**42**	52	36	Rob	PB	3	63	8
**43**	53	26	Rec	PB	3	63	0
**44**	54	42	Rob	PB	9	189	18
**Totals**					283	10837	553

Rec: reciprocal translocation; Rob: Robertsonian translocation; Inv: Inversion; Bla: Blastomere; PB: polar body; TE: trophectoderm cells.

* Chromosomes involved in the rearrangement were not assessed;

** Abnormalities affecting chromosomes involved in the rearrangement were excluded. Case 12 was the only one in which two rearrangements were present. This case was excluded from the statistical analysis.

Of the samples tested from rearrangement carriers, most were abnormal (81.3%) with aneuploidies affecting chromosomes involved in the rearrangement and/or with spontaneously occurring errors affecting unrelated chromosomes. Not surprisingly, the karyotypically normal control group displayed fewer abnormalities (*P*<0.0001), although the rate of aneuploidy was still appreciable; 65.3% of the control samples had an abnormal chromosome number.

In order to assess the presence of an ICE, the chromosomes involved in the rearrangement in each patient were excluded from analysis in both patient and corresponding control groups, thereby focusing the evaluation on chromosomes that were structurally normal in both populations (Table 2 and Table 3). In the samples from the rearrangement carriers, a total of 10,837 chromosomes were examined and 553 aneuploidies were identified. Thus, on average there was a 0.051 probability of a given chromosome having undergone malsegregation. In the control group, 204,406 chromosomes were assessed, leading to the detection of 9,598 distinct abnormalities (0.047 probability of aneuploidy per chromosome per sample). Although small, the increase in the risk of aneuploidy affecting structurally normal chromosomes (0.4% increase per chromosome per sample) was statistically significant (*P*<0.0001), suggesting the existence of an ICE.

**Table 3:** Data concerning the patient-matched control groups.

Matched to case	Age	Stage tested	Number of patients	Embryos with a result	Number of chromosomes assessed*	Number of errors detected**
**1**	26	PB	2	16	336	7
**2**	29	Bla	7	69	2898	136
**3**	30	Bla	5	56	2352	62
**4**	30	Bla	5	56	2352	57
**5**	31	TE	4	27	1134	21
**6**	31	TE	4	27	1134	21
**7**	31	TE	4	27	1134	20
**8**	31	TE	4	27	1134	21
**9**	32	Bla	10	87	3654	169
**10**	32	Bla	10	87	3654	166
**11**	32	TE	5	48	2016	31
**12**	33	Bla	10	93	3906	144
**13**	33	Bla	10	93	3720	140
**14**	33	TE	9	48	2016	27
**15**	34	TE	13	85	3570	44
**16**	34	Bla	18	167	7014	383
**17**	34	Bla	18	167	7014	383
**18**	34	Bla	18	167	7014	380
**19**	34	TE	13	85	3570	46
**20**	35	Bla	17	144	6048	336
**21**	35	Bla	17	144	6048	337
**22**	35	Bla	17	144	6048	291
**23**	35	Bla	17	144	6048	293
**24**	35	TE	8	63	2646	42
**25**	35	TE	8	63	2646	42
**26**	36	TE	13	86	3784	76
**27**	36	Bla	14	120	5040	286
**28**	36	Bla	14	120	5040	282
**29**	36	Bla	14	120	5040	277
**30**	36	Bla	14	120	5040	282
**31**	36	Bla	14	120	5040	282
**32**	36	TE	13	86	3612	73
**33**	36	PB	7	47	987	44
**34**	37	Bla	17	122	5124	289
**35**	37	Bla	17	122	5124	277
**36**	37	Bla	17	122	5124	290
**37**	37	Bla	17	122	5124	276
**38**	38	Bla	16	98	4116	321
**39**	39	Bla	16	108	4536	227
**40**	39	Bla	16	108	4536	240
**41**	40	Bla	16	236	9912	484
**42**	40	Bla	16	236	9912	500
**43**	41	TE	21	120	5040	129
**44**	41	TE	21	120	5040	126
**45**	42	PB	12	65	1365	148
**46**	42	PB	12	65	1365	148
**47**	42	TE	16	76	3192	75
**48**	42	Bla	17	123	5166	388
**49**	43	PB	10	61	1342	137
**50**	43	TE	11	46	2024	96
**51**	36	PB	7	47	987	44
**52**	36	PB	7	47	987	44
**53**	26	PB	2	16	336	8
**54**	42	PB	12	65	1365	150
	Totals			5078	204406	9597

Bla: Blastomere; PB: polar body; TE: trophectoderm cells;

* Chromosomes involved in the matching patient's rearrangement were excluded from the assessed chromosomes.

** Abnormalities affecting chromosomes involved in the matching patient's rearrangement were excluded.

A detailed breakdown of aneuploidies detected at each biopsy stage in patient and control samples is given in Table 4. As expected, samples from rearrangement carriers and samples from the control group both displayed a dramatic increase in aneuploidy with advancing female age. This is presumably due to the well-known increase in meiotic error rate seen as women age. The extent of the ICE was not affected by maternal age, remaining at a similar level for all ages.

**Table 4:** Malsegregation events detected in non-rearranged chromosomes in patient and control samples.

	Patient samples		Control samples	
	Chromosomes	aneuploidies	Chromosomes	aneuploidies
**Polar bodies (mean age 36.6)**	1057	78 (7.4%)	9070	729 (8.0%)
**Blastomeres (mean age 35.4)**	6158	404 (6.6%)	151644	7978 (5.3%)
**Trophectoderm cells (mean age 35.4)**	3622	71 (2%)	43692	890 (2%)
**Total (mean age 35.6)**	10438	524 (5%)	200731	9352 (4.7%)

Although an ICE was apparent when all of the data was summed together, a more detailed assessment, considering each of the different classes of rearrangement separately, showed that not all were consistently associated with elevated chromosome malsegregation. The analysis of results from reciprocal translocation carriers revealed no overall difference in aneuploidy rate compared to the appropriate control group (*P* = 0.87). Some previous findings obtained using FISH analysis of small numbers of chromosomes, mostly carried out in sperm, have suggested that reciprocal translocations can be associated with an ICE [9], [12], while other investigations have reported opposite findings [8], [16], [17]. The current study suggests that most reciprocal translocations are probably not associated with an ICE, but does not rule out the possibility that translocations involving specific breakpoints/chromosomal regions might exhibit this phenomenon.

No ICE was apparent in samples from inversion carriers (*P* = 0.18), however, the number of samples with this class of rearrangement was considered insufficient to conclusively determine whether or not an ICE exists (only 528 chromosomes assessed). The possibility that inversions are associated with an ICE remains unproven and requires further exploration. Interestingly, in the current study, one patient who produced an unusual number of highly abnormal embryos (each embryo affected by multiple aneuploidies) had an inversion in combination with a reciprocal translocation (patient 10 in Table 2). Whether or not the presence of two rearrangements can, together, produce a more potent ICE cannot be determined from a single patient, but remains an intriguing possibility.

Robertsonian translocations were the only class of rearrangement for which an ICE was clearly identified. Comparison of samples from Robertsonian translocation carriers with corresponding control groups revealed a highly significant increase in aneuploidy rate (*P* = 0.0002). However, subdivision of the data revealed that this pronounced ICE was only obvious at the cleavage stage (*P*<0.0001). No ICE was observed in polar bodies (i.e. oocytes) or samples from blastocysts (*P* = 0.72 and *P* = 0.25 respectively). The fact that the ICE was absent from oocytes, but clearly detected in the cleavage stage embryos of both male and female carriers (*P* = 0.0022 and *P* = 0.012 respectively) provides strong evidence for an impact of the rearrangement during mitosis, occurring in the cell divisions immediately following fertilisation. The possibility that some types of translocation might affect the segregation of structurally normal chromosomes during the first few mitotic divisions has not previously been considered, but could explain some of the discordant findings reported in the literature concerning inter-chromosomal effects. Previous studies have tended to be focused on either gametes or embryos, rarely both, and have assumed that any ICE would always have a meiotic origin.

The possibility that early embryogenesis might be particularly sensitive to disruption of chromosome segregation is not without precedent. It is well documented that human embryos display an elevated frequency of chromosomal malsegregation during the first few mitotic divisions following fertilisation. This is true regardless of whether or not the parents have normal karyotypes. The high mitotic error rate frequently results in chromosomal mosaicism and aneuploidy in cleavage stage embryos [25]–[27]. Genetic instability in human preimplantation embryos extends beyond loss/gain of whole chromosomes and also includes chromosome breakage leading to segmental abnormalities in some cases [28]–[30]. The embryonic genome is, for the most part, inactive from fertilization until the 4–8 cell stage and it is thought that a deficiency, or perhaps rigidity, of cell-regulatory mechanisms, including the cell cycle checkpoints that usually act to maintain chromosome segregation and genomic integrity, may be the underlying cause of the observed instability.

Although a developmental phase characterized by genetic instability may provide the necessary environment for errors caused by an ICE to be propagated, it does not explain how the structurally abnormal chromosome disrupts the normal mitotic process. Several studies looking at sperm, have considered the mechanisms that might give rise to an ICE during meiotic divisions [9], [11]. This work has provided an insight into why certain segregation modes are favoured in patients with different types of rearrangement, but the reason why some male translocation carriers display an ICE in their sperm and others do not remains obscure. Similarly, the mechanism by which a rearrangement could disrupt mitosis is not clear. One possibility is that mitotic recombination involving rearranged chromosomes may disturb the ordered arrangement of chromosomes on the spindle, holding together chromosomes that would usually be in separate locations. This is analogous to the principle hypothesis concerning a meiotic ICE, in which the paring of rearranged chromosomes with their structurally normal homologues disrupts the positioning, pairing and segregation of other chromosomes during meiosis [6], [31]. Although mitotic recombination is generally considered to be a rare phenomenon there is good reason to believe it occurs at an appreciable frequency in the cells of human embryos. Mitotic recombination is a mechanism of DNA repair and is usually initiated by a double strand break (DSB). It is becoming increasingly clear that DSBs are very common in the cells of cleavage stage embryos, leading to a high frequency of chromosome breakage [28]–[30]. An elevated incidence of DSBs is further evidenced by the detection of micronuclei, which are often seen in the cells of preimplantation embryos and are suggestive of the presence of fragmented chromosomes.

A second possibility is that the rearranged chromosomes alter normal patterns of chromosome positioning in interphase nuclei, with consequences for attachment to the mitotic spindle during metaphase. The fact that the ICE detected during the current study was only obvious for Robertsonian translocations may be of particular relevance to hypotheses involving positioning in the nucleus or on the spindle. This class of translocations involve acrocentric (D and G group) chromosomes, the short arms of which are composed of tandem copies of ribosomal RNA (rRNA) genes. These regions co-localise during interphase, acting as a focus for formation of the nucleolus. A Robertsonian translocation involves fusion of chromosomes at the centromere, accompanied by loss of the short arms. This is highly likely to lead to the affected chromosomes failing to associate with the nucleolus. Since most chromosomes have specific sequences that interact with nucleoli [32], disturbance of the chromosome territories in this area could have wide ranging effects.

For Robertsonian translocation carriers the risk of any given chromosome being abnormal at the cleavage stage was 0.065. This corresponds to a relative risk of aneuploidy of 1.41 per chromosome compared with matched controls. However, since many embryos contain multiple aneuploidies, the probability of abnormality at the level of the embryo, rather than at the level of the chromosome, is not increased as dramatically. It is inevitable that additional aneuploidies caused by the ICE will often fall within embryos that were already abnormal due to aneuploidy unrelated to the ICE. Of 1,861 embryos in eligible control groups 1,185 (63.68%) were abnormal for one or more chromosomes (excluding embryos in which aneuploidy only affected chromosomes involved in the Robertsonian translocation in the corresponding patient). This compares to a 69.81% aneuploidy rate in the embryos of Robertsonian translocation carriers (again excluding those with abnormality affecting the translocated chromosomes only). Thus, the relative risk of a Robertsonian translocation carrier producing an abnormal cleavage stage embryo, due to an error unrelated to their constitutional rearrangement, is 1.096. In other words, out of every 11 euploid zygotes produced by a Robertsonian translocation carrier one is expected to become abnormal by the cleavage stage due to an ICE.

Most aneuploidies detected in cleavage stage embryos from Robertsonian translocation carriers, which presumably include those arising as a result of the ICE, are of types incompatible with development beyond the first few days of life, producing embryos that would fail to implant or result in an early miscarriage. Indeed, the fact that a significant increase in aneuploidy rate was not seen in blastocyst stage embryos (five days after fertilization of the oocyte) suggests that many of the abnormal embryos, or at least the aneuploid cells potentially produced as a consequence of a mitotic ICE, are rapidly eliminated. The embryonic genome is active during development from the cleavage to the blastocyst stages, which may allow cell-cycle regulatory mechanisms to clear highly abnormal cells via apoptosis or other processes. Nonetheless, it is conceivable that some abnormalities resulting from the ICE could occasionally produce miscarriages or, more rarely, the birth of children with severe congenital abnormalities (e.g. Down, Edwards, Patau, Klinefelter or Turner syndromes).

While the incidence of aneuploidy at birth is only likely to be slightly elevated, there may be a more pronounced effect on fertility, due to the increased mortality of early embryos. This may be particularly relevant for patients using assisted reproductive techniques such IVF or PGD. An important clinical consideration is that for Robertsonian translocation carriers undergoing IVF, preimplantation genetic diagnosis using a comprehensive chromosome screening technique, capable of detecting aneuploidy affecting any chromosome is highly advisable [21]–[24], [33]–[36]. Older PGD methods utilizing FISH, which focus on analysis of the rearranged chromosomes only, should be discouraged, since they will fail to detect abnormalities affecting other chromosomes, arising as a consequence of an ICE.

In conclusion, an ICE affects carriers of Robertsonian translocations and may contribute to higher rates of abnormal embryos, resulting in a small increase in the risk of miscarriage and birth of children with congenital abnormalities and a potential reduction in fertility. These possibilities should be considered when counselling patients about the risk of abnormal pregnancy following natural conception or the likelihood of producing embryos suitable for uterine transfer during cycles of PGD. Further research to determine the mechanism by which rearranged chromosomes can disrupt the process of mitotic chromosome segregation should be encouraged. In the case of cleavage stage embryos, the combination of chromosome rearrangement and compromised cellular mechanisms for the maintenance of genetic fidelity may be important. Chromosome rearrangements and reduced genetic stability are also a hallmark of many tumor cells, suggesting that the mitotic ICE described here may have important implications in cancer research and the understanding of tumor evolution as well as in developmental biology and clinical genetics.

## Materials and Methods

All patients had their chromosomal rearrangements accurately defined by clinical cytogeneticists using standard chromosome banding techniques. The samples were obtained during 54 PGD cycles undertaken for 44 couples (Table 1). The average maternal age was 35.6 years (maternal age range 26–43) and the chromosome rearrangements included 20 Robertsonian translocations, 23 reciprocal translocations and 3 inversions (for one couple, the male partner was a carrier of a reciprocal translocation and the female partner was a carrier of an inversion). The PGD cases were undertaken over a three year period, with patients undergoing assisted reproductive treatment at seven different IVF clinics and genetic diagnosis performed at two different PGD laboratories (Reprogenetics UK [Oxford, UK] and Reprogenetics LLC [New Jersey, USA]). All IVF centres involved in this study had the necessary ethical and clinical approvals and licenses required for the tests offered to patients. All patients were provided with counselling regarding PGD using aCGH, and signed consent was obtained in all cases.

Diagnosis was performed at one of three different preimplantation stages (Table 1). All biopsies involved breach of the zona pellucida encapsulating the oocyte/embryo with a laser, regardless of the embryonic stage being tested. Standard methods of ovarian stimulation, biopsy and embryo culture were utilized. Oocyte analysis involved aCGH of the first and second polar bodies; cleavage stage analysis was undertaken with the biopsy of a single blastomere three days after fertilization of the oocyte (8-cell stage); blastocyst evaluation employed removal of approximately five cells from the trophectoderm layer five days after fertilization.

Microarray-CGH analysis was undertaken according to our previously validated protocol [21]. Briefly, whole genome amplification was carried out in order to generate sufficient DNA from the minute samples under analysis (SurePlex, Rubicon, USA). Amplified sample and reference DNAs were labelled with Cy3 and Cy5 respectively and then hybridized to the probes of a bacterial artificial chromosome (BAC) microarray (24Sure+, BlueGnome, Cambridge, USA). Chromosome losses and gains were revealed by differences in the fluorescence intensity corresponding to sample and reference DNAs for BAC probes derived from the affected chromosome or chromosomal region. Labelling of the amplified samples, hybridization to microarray slides, post-hybridization washes and analyses were performed as described previously [21]. Published values for the accuracy rate for aCGH are 94%, 98% and 95% for polar bodies, blastomeres and trophectoderm cells, respectively [36]–[38].

In order to assess whether the incidence of abnormalities was the same in the presence of a chromosome rearrangement, the data from patient and control samples were compared statistically (Chi squared test with Yates' correction). However, pooling data from multiple patient-specific controls was not straightforward, as some control groups were composed of larger numbers of samples than others. Given the importance of female age in aneuploidy predisposition the control data could have been inadvertently skewed by including disproportionately large numbers of samples from individuals of specific ages. To overcome this problem, we ensured that the samples from each patient-specific control were given equal weight to the samples from the matched patient (i.e. control data was transformed to match the sample number of the corresponding patient). In order to accomplish this we first deduced the frequency of aneuploid chromosomes per sample in each control group (i.e. total number of errors divided by total number of chromosomes analysed). We then multiplied this figure by the number of chromosomes assessed for the corresponding matched patient (after excluding the chromosomes involved in the rearrangement). The result represents the number of chromosome errors that would have been expected in the control group had it consisted of the same number of samples as produced by the matched patient. The expected numbers of aneuploidies for the control groups are detailed in Table 5.

**Table 5:** Corrected number of errors in the patient-specific control groups.

Case	Age	Number of patient samples	Number of matched control samples	Number of chromosomes assessed in control*	Number of errors detected in control**	Expected number of errors***	Expected number of normal chromosomes***
**1**	26	3	16	336	7	1	62
**2**	29	7	69	2898	136	14	280
**3**	30	11	56	2352	62	12	450
**4**	30	5	56	2352	57	5	205
**5**	31	13	27	1134	21	10	536
**6**	31	9	27	1134	21	7	371
**7**	31	3	27	1134	20	2	124
**8**	31	2	27	1134	21	2	82
**9**	32	4	87	3654	169	8	160
**10**	32	5	87	3654	166	10	200
**11**	32	7	48	2016	31	5	289
**12**	33	2	93	3906	144	3	81
**13**	33	8	93	3720	140	12	308
**14**	33	9	48	2016	27	5	373
**15**	34	11	85	3570	44	6	456
**16**	34	3	167	7014	383	7	119
**17**	34	7	167	7014	383	16	278
**18**	34	3	167	7014	380	7	119
**19**	34	5	85	3570	46	3	207
**20**	35	2	144	6048	336	5	79
**21**	35	5	144	6048	337	12	198
**22**	35	3	144	6048	291	6	120
**23**	35	7	144	6048	293	14	280
**24**	35	5	63	2646	42	3	207
**25**	35	4	63	2646	42	3	165
**26**	36	3	86	3784	76	3	129
**27**	36	3	120	5040	286	7	119
**28**	36	2	120	5040	282	5	79
**29**	36	4	120	5040	277	9	159
**30**	36	5	120	5040	282	12	198
**31**	36	8	120	5040	282	19	317
**32**	36	2	86	3612	73	2	82
**33**	36	4	47	987	44	4	80
**34**	37	6	122	5124	289	14	238
**35**	37	5	122	5124	290	12	198
**36**	37	9	122	5124	276	20	358
**37**	37	6	122	5124	277	14	238
**38**	38	2	98	4116	321	7	77
**39**	39	8	108	4536	227	17	319
**40**	39	2	108	4536	240	4	80
**41**	40	7	236	9912	484	14	280
**42**	40	3	236	9912	500	6	120
**43**	41	3	120	5040	129	3	123
**44**	41	5	120	5040	126	5	205
**45**	42	9	65	1365	148	20	169
**46**	42	8	65	1365	148	18	150
**47**	42	3	76	3192	75	3	123
**48**	42	5	123	5166	388	16	194
**49**	43	7	61	1342	137	16	138
**50**	43	2	46	2024	96	4	84
**51**	36	4	47	987	44	4	80
**52**	36	3	47	987	44	3	60
**53**	26	3	16	336	8	2	61
**54**	42	9	65	1365	150	21	168
		283	5078	204406	9598	462	10375

* Chromosomes involved in the matching patient's rearrangement were excluded from the assessed chromosomes.

** Abnormalities affecting chromosomes involved in the matching patient's rearrangement were excluded.

*** Expected number in the control group if it consisted of the same number of samples as provided by the matched patient.
