# Correction: Embryos of Robertsonian Translocation Carriers Exhibit a Mitotic Interchromosomal Effect That Enhances Genetic Instability during Early Development

**DOI:** 10.1371/journal.pgen.1010377

**Published:** 2022-08-29

**Authors:** Samer Alfarawati, Elpida Fragouli, Pere Colls, Dagan Wells

There are a number of errors in the caption for Fig 1, “Microarray-CGH analysis of an embryo from a Robertsonian translocation carrier.” Please see the complete, correct Fig 1 caption here.

**Fig 1:**
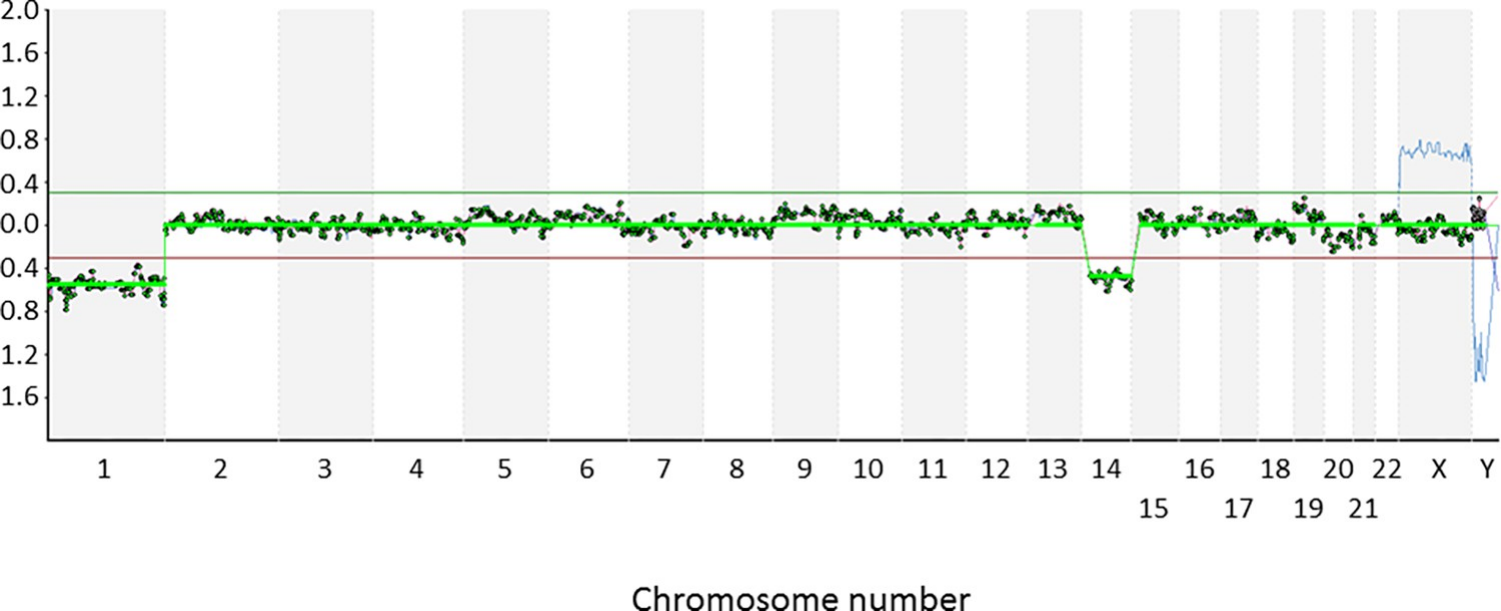
Microarray-CGH analysis of an embryo from a Robertsonian translocation carrier. Cytogenetic analysis of a cleavage stage embryo from a Robertsonian translocation carrier- 46,XY,t(13;14). Microarray-CGH revealed monosomy 14, presumably resulting from a meiotic error due to problems processing the rearranged chromosomes. An additional aneuploidy unrelated to the Robertsonian translocation (monosomy for chromosome 1) was also detected. The two monosomies are indicated by the altered ratio of fluorescence related to the test (embryo) and reference (46,XY) DNA samples. All of the probes corresponding to chromosomes 1 and 14 have test/reference ratios less than 0.3.
